# Giant ovarian tumor with colorectal cancer: suggestion concerning the need for colonoscopy screening in cases with large ovarian tumor—a report of three cases

**DOI:** 10.1186/s12893-022-01565-4

**Published:** 2022-03-23

**Authors:** Yoshiaki Maeda, Nozomi Minagawa, Hirotaka Shoji, Tadayuki Kobayashi, Keiichiro Yamamoto

**Affiliations:** Department of Gastrointestinal Surgery, Hokkaido Cancer Center, Sapporo, Japan

**Keywords:** Colorectal cancer, Ovarian metastasis, Ovarian carcinoma, Giant ovarian tumor, Colonoscopy

## Abstract

**Background:**

Patients with giant ovarian tumor often have severe symptoms, such as abdominal distention, and the tumor tends to grow rapidly; therefore, sufficient preoperative assessments are difficult to perform. It is not always easy to differentiate between primary and metastatic ovarian cancer, especially when the ovarian tumor is huge, since a precise diagnosis of ovarian tumor depends on the histopathological findings of the excised specimen. Although metastatic ovarian tumors account for over 20% of all malignant ovarian tumors, preoperative colonoscopy is not considered a routine examination before surgery for giant ovarian tumor.

**Case presentation:**

We herein report 3 cases of giant (> 25 cm) ovarian tumor with colorectal cancer. All three patients visited the clinic with progressing abdominal distention, and were referred with primary ovarian malignancy. *Case 1:* Rectal tumor was suspected by a digital examination at the outpatient clinic, and rectal cancer was diagnosed preoperatively by colonoscopy. Computed tomography revealed a single-nodule liver tumor. Ovariectomy, rectal resection, and partial hepatectomy were performed. A histological examination revealed both primary mucinous ovarian carcinoma and rectal carcinoma with liver metastasis. *Case 2:* Initially, the ovarian tumor was diagnosed as primary carcinoma based on the histological findings of an incision biopsy at the previous hospital. Chemotherapy for ovarian cancer was administered without remission, and subsequently, the patient was referred to our hospital. Since the CEA level was high (142 ng/ml), colonoscopy was performed and cecal cancer was diagnosed. Ovariectomy and right colectomy were performed, and the ovarian tumor was histologically diagnosed as metastatic adenocarcinoma. *Case 3:* Initial ovariectomy was performed, and rectal cancer was suspected at intra-operative surveillance. Colonoscopy was performed after surgery, and rectal cancer was diagnosed. The ovarian tumor was diagnosed as metastatic adenocarcinoma. After six cycles of FOLFOX, rectal resection was performed.

**Conclusion:**

Regrettably, two of three cases in the current series were not diagnosed with colorectal cancer at the start of treatment. This experience suggests that screening colonoscopy should be considered before treatment for every case of giant ovarian tumor.

## Background

Giant ovarian tumor includes not only primary ovarian cancer but also metastatic malignancy, such as that originating from colorectal cancer. Metastatic ovarian tumors account for over 20–30% of all malignant ovarian tumors [1, 2]. In recent reports, colorectal cancer accounted for 65% of ovarian metastases, with an increased percentage of cases in recent years [3]. Metastatic colorectal cancer may have the same presentation as advanced ovarian cancer, including pelvic mass and ascites [4]. A preoperative diagnosis between primary ovarian cancer and metastatic tumor is important to determine treatment strategies, but often challenging and difficult [5]. Despite advancements in imaging modalities, including computed tomography (CT), positron emission tomography (PET), ultrasonography (US), and magnetic resonance imaging (MRI), the final diagnosis of ovarian malignancy depends on a histological examination, particularly the findings of immunohistochemical staining [6].

Colonoscopy is the most sensitive examination for evaluating the presence of colorectal malignancy; however, screening colonoscopy is not considered a required preoperative investigation for primary ovarian cancer [5, 7]. In actual clinical practice, patients with metastatic ovarian cancer originating from colorectal cancer often undergo surgery based on a misdiagnosis of primary ovarian malignancy.

We herein report three cases of giant ovarian carcinoma with colorectal carcinoma, two of which were metastatic ovarian carcinoma and the other was primary ovarian cancer.

## Case presentation

Below are described three cases of giant (> 25 cm) ovarian tumor with colorectal cancer. All three patients visited the clinic with progressing abdominal distention, and were referred with primary ovarian malignancy. The detailed profiles of the patients are described in Table 1.

**Table 1:** Detailed feature of the patients

Case	Age	Chief compliant	Preexisting conditions	CT findigs of ovarian tumor	CEA (ng/ml)	CA19-9 (U/ml)	CA125 (U/ml)	Hb (g/dl)	Histology of ovarian tumor	IHC findings of ovarian tumor	Feature of colorectal cancer	Diagnosis of colorectal cancer
1	53	Abdominal distention	None	34 × 29 cm Cystic mass with irregularly shaped solid component	8.6	2687	208	8.8	Mucinous carcinoma, ovarian primary	CK7 + , CK20− CDX2-	Ra 75 × 50 mm type2 tub2 pT3 N2 M1 (liver)	Digital examination at outpatient clinic
2	58	Abdominal distention, lower abdominal pain	None	34 × 28 cm Cystic mass with irregularly shaped septum and solid component	142	117	328	9.8	Metastatic adenocarcinoma	CK7− CK20 + CDX2 + ER−	C 35 × 30 mm type2 tub2 pT4a N0	Colonoscopy (3 months after start of chemotherapy)
3	61	Abdominal distention, severe constipation	Diabetes, hyperthyroidism	29 × 26 cm Polycystic mass with irregularly shaped septum fed by left ovarian artery	10.0	2546.0	269	11.0	Metastatic adenocarcinoma	CK7−, CK20 + , CDX2 + , ER−	Rs 54 × 45 mm type2 tub1 pT3 N0	Intra-operative survey during ovariectomy

*Hb* hemoglobin, *IHC* immunohistochemical

### Case 1

**Fig. 1:**
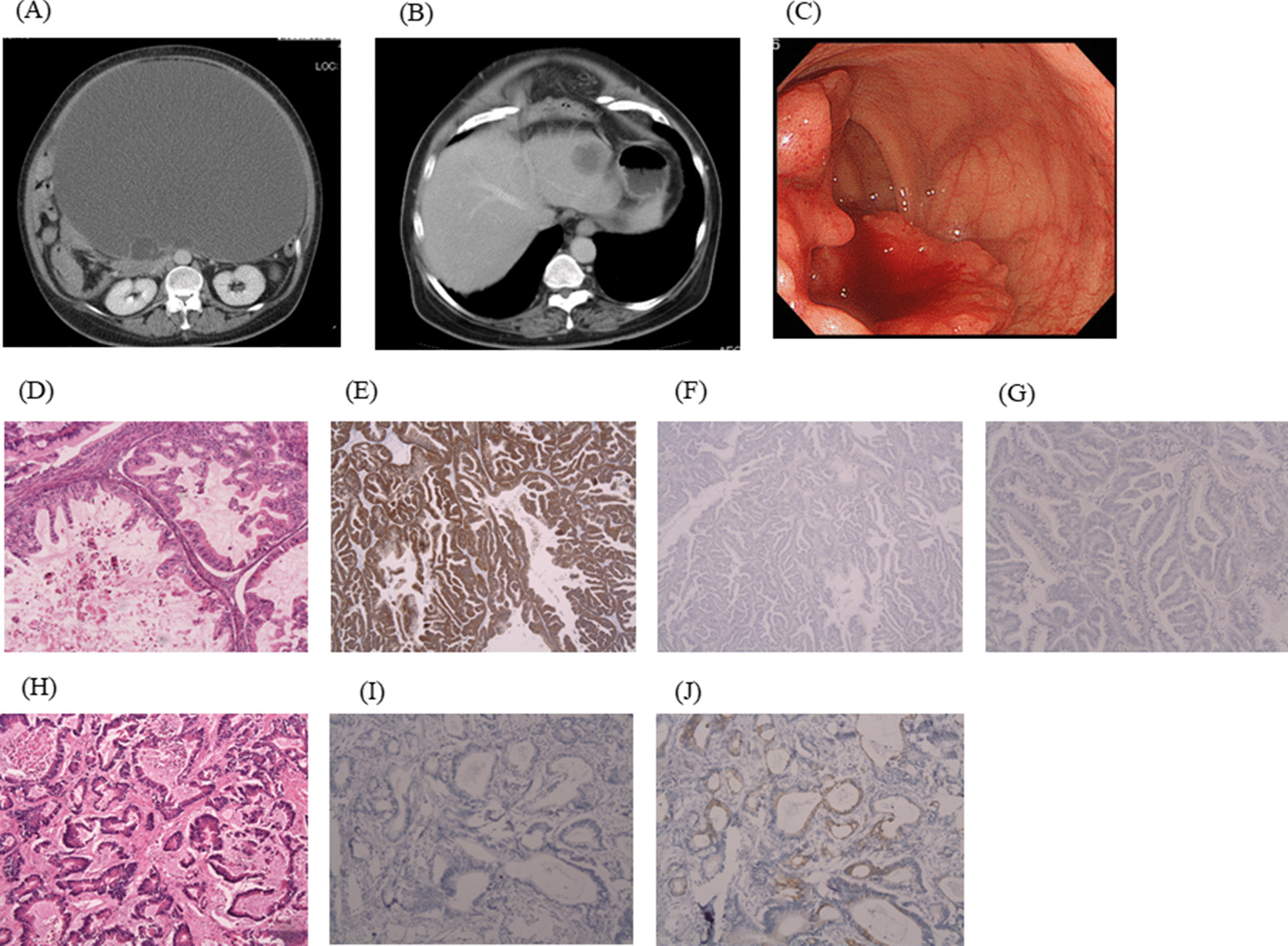
**A**, **B** Enhanced CT revealed a cystic ovarian mass with an irregularly shaped solid component measuring 34 × 29 cm and single-nodule liver tumor measuring 3.8 cm in segment 2. **C** Colonoscopy showed type 2 cancer in the lower rectum. **D** A histopathological evaluation of the ovarian tumor showed mucinous adenocarcinoma forming a cystic lesion containing mucin (Hematoxylin and eosin (HE) 100×). Immunohistochemistry staining showed that the ovarian tumor was CK7-positive (**E**), CK20-negative (**F**), and CDX2-negative. (**H**) Histopathological findings of the liver tumor showed adenocarcinoma consisting of atypical columnar epithelium with necrosis (HE 200×). (**G**) Immunohistochemistry staining showed that the liver tumor was CK7-negative (**I**) and CK20-positive (**J**)

A 53-year-old woman was referred to the gynecology department with a 2-month history of increasing abdominal distention. Enhanced CT showed a cystic ovarian mass with an irregularly shaped solid component measuring 34 × 29 cm (Fig. 1A). CT also revealed a 38 mm single-nodule liver tumor in segment 2 (Fig. 1B). A rectal tumor was suspected based on a digital examination at the outpatient clinic, and lower rectal cancer was diagnosed preoperatively by colonoscopy (Fig. 1C). Ovariectomy, abdominoperineal rectal excision, and partial hepatectomy were performed. A histopathological evaluation of the ovarian tumor showed mucinous adenocarcinoma forming cystic lesions containing mucin (Fig. 1D). Immunohistochemistry staining showed that the ovarian tumor was CK7-positive (Fig. 1E), CK20-negative (Fig. 1F) and CDX2-negative (Fig. 1G). The histopathological findings of the liver tumor showed adenocarcinoma consisting of atypical columnar epithelium with necrosis (Fig. 1H). Immunohistochemistry staining showed that the liver tumor was CK7-negative (Fig. 1I) and CK20-positive (Fig. 1J). Primary mucinous ovarian carcinoma and rectal carcinoma with liver metastasis were diagnosed.

### Case 2

**Fig. 2:**
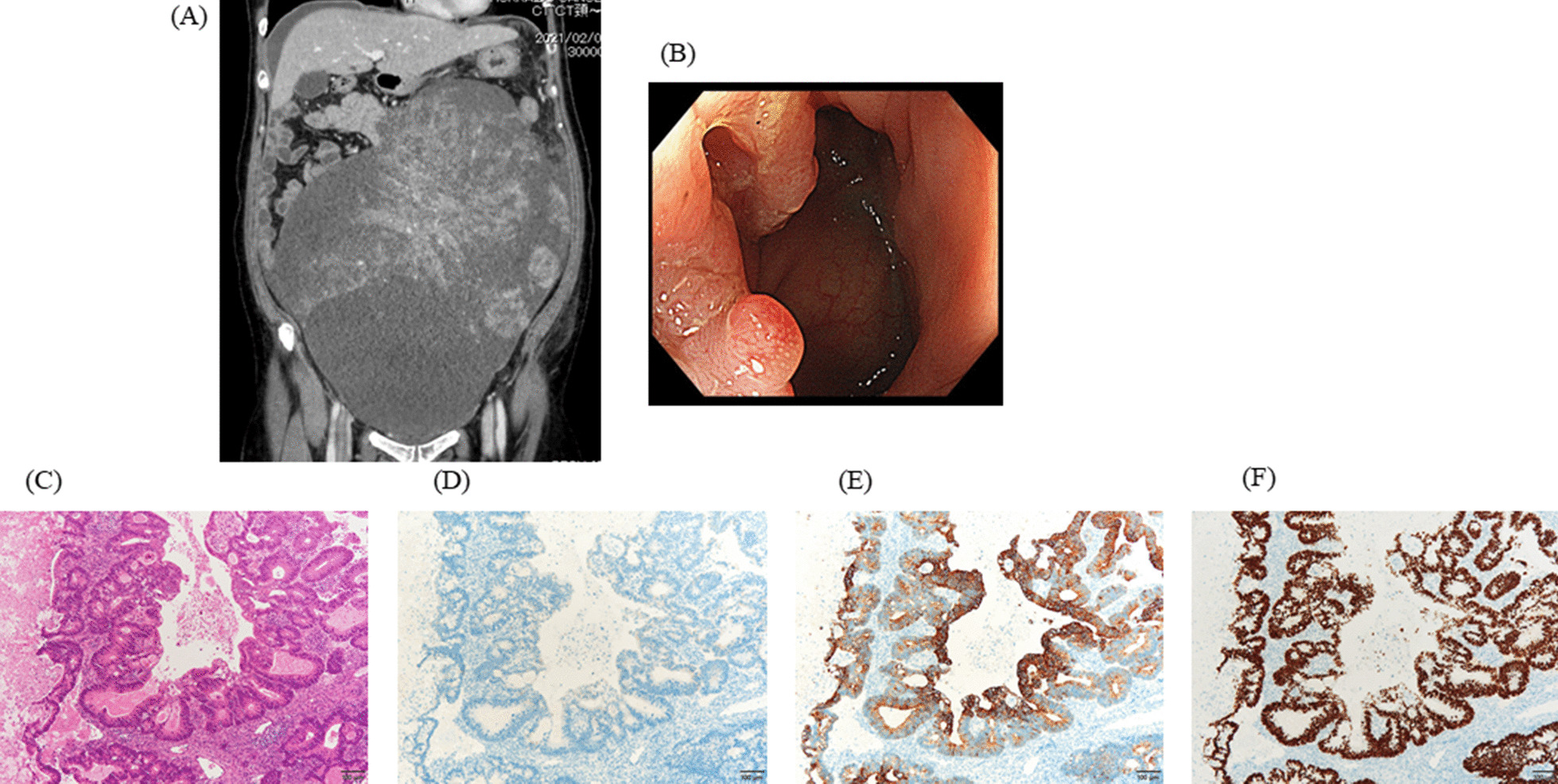
**A** Enhanced CT revealed a cystic ovarian mass with an irregularly shaped septum and a solid component measuring 34 × 28 cm. **B** Colonoscopy showed type 2 cecal cancer. **C** A histopathological evaluation of the ovarian tumor showed adenocarcinoma consisting of atypical columnar epithelium with severe necrosis (HE 200×). Immunohistochemistry staining showed that the ovarian tumor was CK7-negative (**D**), CK20-positive (**E**), and CDX2-positive (**F**)

A 58-year-old woman was referred to a hospital with lower abdominal pain. Enhanced CT revealed a cystic ovarian mass with an irregularly shaped septum and solid component measuring 34 × 28 cm (Fig. 2A). Initially, the ovarian tumor was diagnosed as primary carcinoma based on histological findings of a specimen from an incision biopsy. Chemotherapy for ovarian cancer was administered without remission, and subsequently, the patient was referred to our hospital. Since the CEA level was high (142 ng/ml), colonoscopy was performed, and cecal cancer was diagnosed (Fig. 2B). Ovariectomy and right colectomy were performed, and the ovarian tumor was histologically diagnosed as adenocarcinoma consisting of atypical columnar epithelium with severe necrosis (Fig. 2C). Immunohistochemistry staining showed that ovarian tumor was CK7-negative (Fig. 2D), CK20-positive (Fig. 2E), and CDX2-positive (Fig. 2F), suggesting metastasis of cecal cancer.

### Case 3

**Fig. 3:**
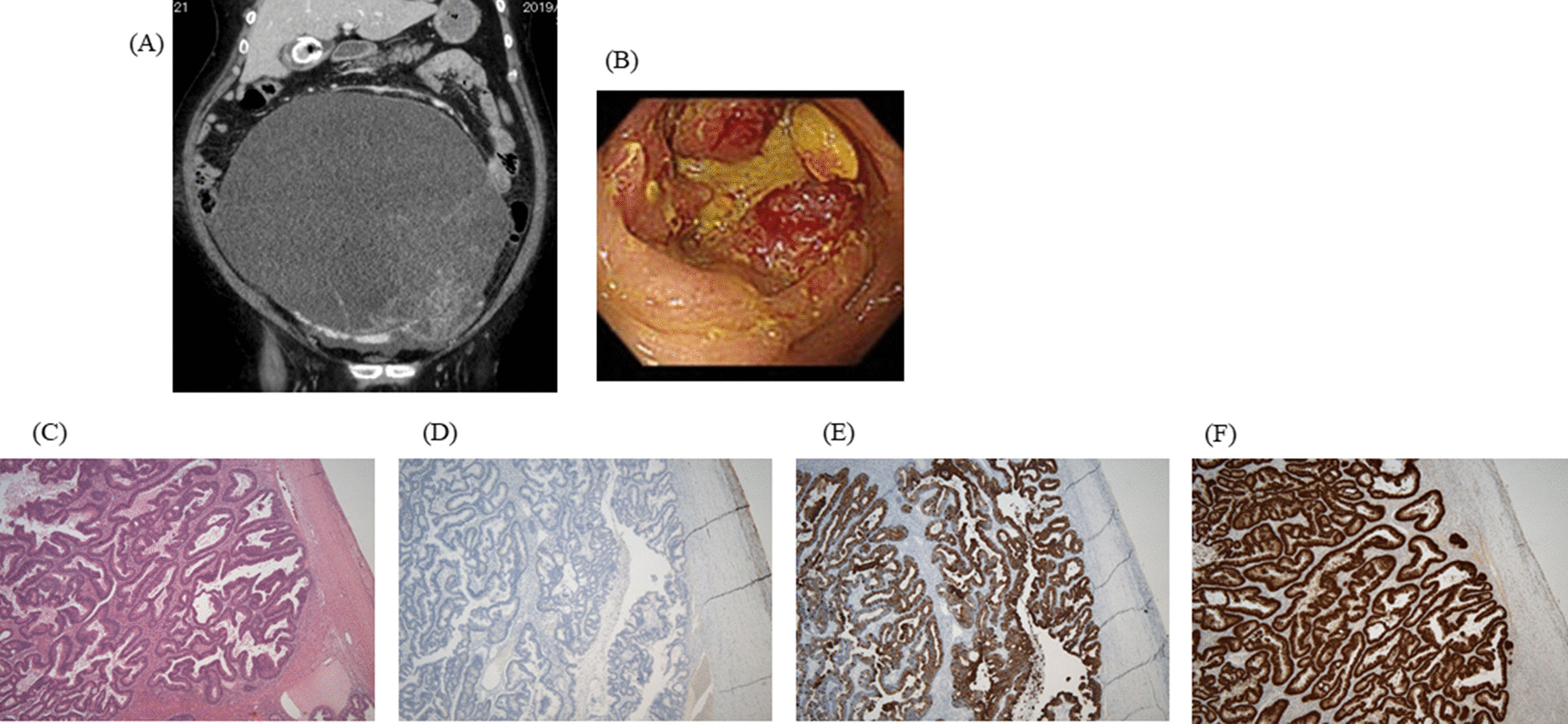
**A** Enhanced CT showed 29 × 26 cm polycystic mass with an irregularly shaped septum, fed by the left ovarian artery. **B** Colonoscopy after ovariectomy showed type 2 sigmoid colon cancer. **C** A histopathological evaluation of the ovarian tumor showed adenocarcinoma consisting of atypical stratified columnar epithelium with necrosis (HE 100×). Immunohistochemistry staining showed that the ovarian tumor was CK7-negative (**D**), CK20-positive (**E**), and CDX2-positive (**F**)

A 61-year-old woman was referred to our hospital with progressive abdominal distension and severe constipation. Enhanced CT showed 29 × 26 cm polycystic mass with an irregularly shaped septum, fed by the left ovarian artery (Fig. 3A). Ovariectomy was performed, and rectal cancer was suspected at intra-operative surveillance. Colonoscopy was performed after surgery, and rectal cancer was diagnosed (Fig. 3B). A histopathological evaluation of the ovarian tumor showed adenocarcinoma consisting of atypical stratified columnar epithelium with necrosis (Fig. 3C). Immunohistochemistry staining showed that the ovarian tumor was CK7-negative (Fig. 3D), CK20-positive (Fig. 3E), and CDX2-positive (Fig. 3F). The ovarian tumor was diagnosed as metastatic adenocarcinoma. After six cycles of FOLFOX, rectal resection was performed.

## Discussion and conclusions

We encountered three cases of giant ovarian tumor with coexisting colorectal cancer. Two of them had metastatic ovarian carcinoma of colorectal origin, and the other had primary ovarian cancer. Regrettably, two of the three cases had not been diagnosed with colorectal cancer at the start of treatment.

Making a preoperative diagnosis of ovarian tumor is often challenging and difficult, since patients with giant ovarian tumor include not only those with primary ovarian carcinoma but also those with metastatic tumor and pathologically benign tumor mimicking malignancy [8–10]. Metastatic ovarian tumors have been reported to account for over 20–30% of all malignant ovarian tumors [1, 2]. Colorectal cancer account for 65% of ovarian metastases, with an increasing percentage reported in recent years [1, 3, 11]. Conversely, ovarian metastases occur in 5–10% of women with metastatic colorectal cancer [12]. Most of the giant ovarian tumors reported were more than 25 cm in diameter [6, 9, 10, 13]. A pre-treatment differential diagnosis between primary ovarian cancer and metastatic tumor is more difficult when the ovarian tumor is huge [5], since severe symptoms such as abdominal distention and progressive tumor growth, may hinder further examinations and limit the time for a preoperative assessment. Some radiologists claim that a mixed cystic and solid ovarian mass should be regarded as a metastatic tumor, especially in patients with a history of colorectal cancer [14]; however, other authors insist that depending on radiographic studies is inadequate for differentiating between primary and metastatic ovarian tumors [12]. Presently, a precise diagnosis of ovarian tumor depends on the histopathological findings of excised specimen. An immunohistochemical evaluation is essential for distinguishing between primary and metastatic ovarian carcinoma [6]. Colorectal carcinomas are generally negative for CK7 but positive for CK20 and CDX2, whereas primary ovarian cancers are mostly (> 90%) positive for CK7 and negative for CK20 and CDX2 [11].

Although resection of malignant ovarian tumor can provide a survival benefit for both primary ovarian carcinoma and metastatic ovarian carcinoma originating from colorectal cancer, the operative procedures differ greatly, depending on whether the case is one of primary or metastatic ovarian cancer. Extended surgery, including hysterectomy, omentectomy, and lymph node dissection is needed for primary ovarian cancer surgery [15]. Furthermore, neoadjuvant chemotherapy, is often administered prior to surgery for ovarian cancer [2, 16]. Therefore, pre-treatment detection of colorectal cancer is crucial for deciding on a treatment strategy, so adequate chemotherapy regimens should be chosen depending on the primary tumor [17]. Case 2 in the present study was initially misdiagnosed as primary ovarian carcinoma based on the histological findings of an incision biopsy made at the previous hospital without an immunohistochemical study. Screening colonoscopy was not performed despite the elevated serum CEA level. As a result, neoadjuvant chemotherapy for ovarian cancer was mistakenly administered, without remission. The patient was treated improperly for 6 months before she was referred to our hospital and underwent colonoscopy. These facts indicate that an adequate diagnosis at the start of treatment is essential for achieving the best treatment outcomes.

Regrettably, two of the three cases in the current case series had not been diagnosed with colorectal cancer at the start of treatment. Although colonoscopy is a gold standard in evaluating the presence of colorectal malignancy, screening colonoscopy is not considered required as a preoperative investigation for primary ovarian cancer [5, 7]. As a result, in actual clinical practice, patients with metastatic ovarian cancer originating from colorectal cancer often undergo surgery based on a misdiagnosis of primary ovarian malignancy. Saltzman et al. reported that 5 of 212 (2%) gynecologic oncology patients had been diagnosed with colorectal cancer at pre-treatment screening colonoscopy; however, they concluded that colon screening was not necessary in the preoperative workup of gynecologic oncology patients [7]. Renaud et al. reported that 7% had a primary GI cancer in their case series of 71 ovarian malignancies [3]. Ravizza et al. concluded that colonoscopy identified a not insignificant number of patients requiring colorectal surgery. In their prospective study of 144 consecutive patients with a supposed primary ovarian cancer, 6 (4%) patients were diagnosed with colorectal cancer metastatic to the ovary. Furthermore, 8 (6%) patients were diagnosed with bowel infiltration at screening colonoscopy [18]. Preoperative computed tomography dedicated to examining the bowel may be a viable alternative to colonoscopy, but not completely [8]. Given that colon cancer is by far more frequent than ovarian carcinoma, screening colonoscopy should be considered necessary in every case of giant ovarian tumor before treatment.

This case series demonstrates that screening colonoscopy should be considered routinely before treatment for cases of giant ovarian tumor, and multidisciplinary approach is important in order to make the right diagnosis and offer the best treatment.

## Data Availability

All data related are included in the manuscript.

## Abbreviations

CT: Computed tomography
PET: Positron emission tomography
US: Ultrasonography
MRI: Magnetic resonance imaging
CK7: Cytokeratin 7
CK20: Cytokeratin 20
CDX2: Caudal-related homeobox transcription factor 2
CEA: Carcinoembryonic antigen
CA19-9: Carbohydrate antigen 19-9
CA125: Carbohydrate antigen 125

## Declarations

### Limitations

This study was a report of only three cases in a single center, therefore, was not able to show the proportion of metastatic or primary cancer among the giant ovarian tumor.
